# Rosacea fulminans: unusual clinical presentation of rosacea*

**DOI:** 10.1590/abd1806-4841.20164943

**Published:** 2016

**Authors:** Jessica Castiel Coutinho, Danielle Cristine Westphal, Laís Cruz Lobato, Antônio Pedro Mendes Schettini, Mônica Santos

**Affiliations:** 1 Dermatologist – Manaus (AM), Brazil; 2 Municipal Secretary of Health of Amazonas – Manaus (AM), Brazil; 3 Fundação Alfredo da Matta (Fuam) – Manaus (AM), Brazil; 4 Universidade do Estado do Amazonas (UEA) – Manaus (AM), Brazil

**Keywords:** Rosacea, Clinical Symptoms, Therapeutics

## Abstract

Rosacea fulminans or pyoderma faciale is a rare cutaneous disorder that usually affects women usually between the ages of 15-46. The disease is characterized by sudden onset of papules, pustules, cysts, and painful coalescing nodules with red-cyanotic centrofacial erythema. Although its etiology remains unknown, hormonal, immunological, and vascular factors have been reported. Early diagnosis and prompt treatment should minimize unsightly scars. We report a case of a 33-year-old female patient treated with traditional doses of doxycycline, with improvement of the lesions and regression of the condition in two months.

## INTRODUCTION

Rosacea fulminans, also known as facial pyoderma, is a rare condition, considered as an exacerbated form of rosacea. Although it occurs mostly in women aged 15-46, there are sporadic reports in men and children. Its cause remains unknown. However, it is believed that hormones contribute to the development of the lesions since the condition is much more common in females and, in some cases, has been associated with pregnancy.^1^ It is rarely associated with other diseases, such as inflammatory bowel disease, thyroid disease, and liver disease.^1,2,3^ Treatment options include corticosteroids, isotretinoin, dapsone, and antibiotics.^1,2,4^

## CASE REPORT

**Figure 1:**
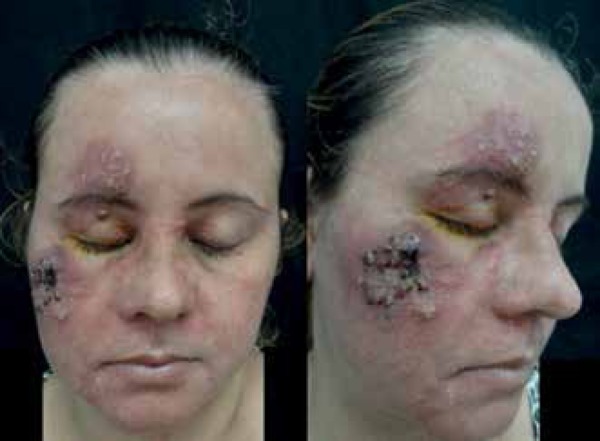
Erythematous edematous plaques on the right hemifacial area with inflammatory nodules, pustules, and extensive area of necrosis. Accentuated seborrhoeic dermatitis on the face and absence of comedones

**Figure 2:**
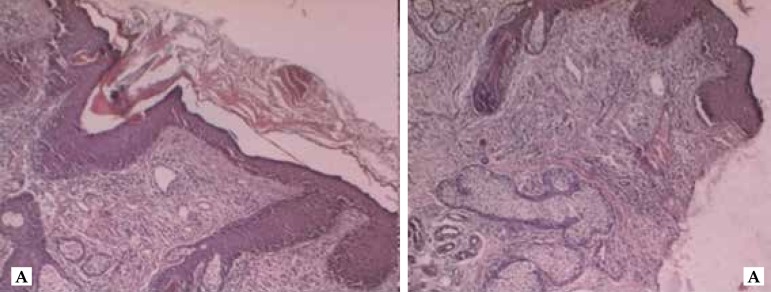
Epidermis shows hyperkeratosis with follicular plugging and rectification of interpapillary cones. Papillary dermis showed dilated vessels and hair follicles surrounded by a mixed inflammatory process, consisting of neutrophils and lymphocytes in periadnexal and perivascular disposition. HE 5x

**Figure 3:**
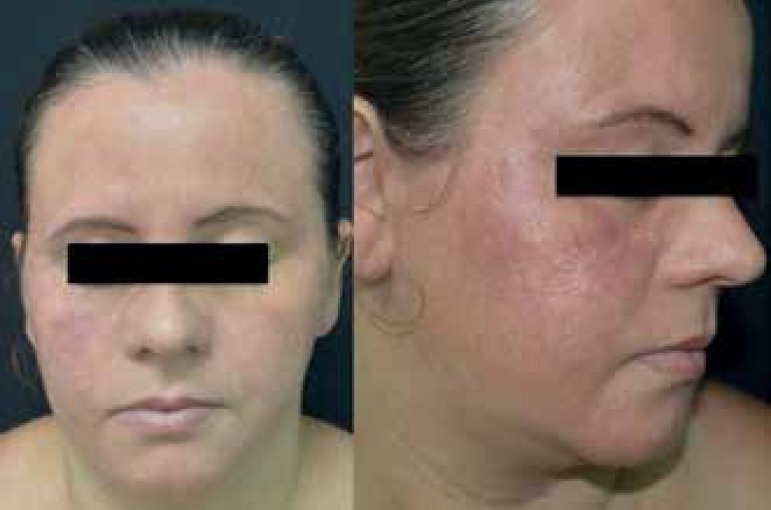
Presence of erythema and telangiectasia, with regression of the edema, pustules, and necrosis

We report a 33-year-old female patient with a diagnosis of psoriasis vulgaris receiving irregular follow-up. The patient reported the abrupt appearance of painful lesions on the face 15 days before. Physical examination revealed erythematous and edematous plaques on the right hemifacial area with inflammatory nodules, pustules, and an extensive area of necrosis. We also observed severe seborrhea on the face and absence of comedones (Figure 1). Histopathological examination with hematoxylin-eosin stain showed hyperkeratosis in the epidermis with the presence of follicular plugging and rectification of the interpapillary cones. Papillary dermis showed dilated vessels and hair follicles surrounded by a mixed inflammatory process, consisting of neutrophils and lymphocytes (Figure 2). Considering this clinical features, we diagnosed rosacea fulminans and started treatment with doxycycline 200 mg/day. After two months of treatment, we observed a significant improvement, with regression of edema, pustules, and necrosis, but with persistent erythema and telangiectasias (Figure 3). Patient remains in treatment with metronidazole 1% with good clinical management.

## DISCUSSION

Described in 1940 by O’Leary and Kierland, rosacea fulminans was originally called pyoderma faciale and believed to be a variant of acne conglobata. It was only in 1992 when Plewig et al. suggested it to be a severe variant of rosacea (not a variant of acne) naming the disease rosacea fulminans.^4^ Its etiology has not been fully elucidated. However, it is believed that hormones contribute to the development of the lesions due to a higher prevalence in women and case reports in pregnant women. Patients with rosacea displayed immunoglobulins in the dermal-epidermal junction, increased innate immune response, and activation of the adaptive immune response with an increase of cathelicidin LL-37, kallikrein 5, and interleukin-8.^1,3,5^ Many believe that rosacea may be a vascular disorder because of its association with vasodilation, increased cutaneous blood flow, and vascular permeability. The results are fluid leakage with consequent tissue inflammation and an increase of growth factors that stimulate angiogenesis.^5^ Some cases report the association of pyoderma faciale with inflammatory bowel disease, thyroid disease, and liver disease. In addition, the use of high doses of vitamins B6 and B12, pegylated interferon, and ribavirin may act as possible triggers.^1,2,3^ The clinical features are sudden onset of painful coalescing papules, pustules, cysts, and nodules, combined with red-cyanotic erythema, usually restricted to the face, especially in the centrofacial region. Extrafacial eruption – posterior neck, breasts, shoulders, and extremities – has been described less frequently. Ocular involvement may be present, which is often an important sign for the diagnosis of rosacea fulminans. Other signs reported include hyperemia, foreign body sensation, dry eye, and blurred vision. As in the present case, the presence of severe seborrhea is observed in most cases.^6^ Patients are previously healthy and there is no impairment of general condition. Constitutional symptoms are rare and characterized by weight loss, fatigue, tiredness, discomfort, malaise, and fever. Previous history of acne is present in less than 50% of cases.^3^ Diagnosis is based on clinical history, physical examination, laboratory tests, and histopathology. Laboratory findings are nonspecific and include mild anemia, mild leukocytosis, increased erythrocyte sedimentation rate, and elevated Creactive protein.^7^ Bacterial culture of purulent material is negative in most cases, but can reveal *Staphylococcus epidermidis*, *Corynebacterium sp*., *Propionibacterium acnes*, *Streptococcus viridans*, and gram negative bacteria – such as Enterobacter cloacae and Klebsiella oxytoca.^7,8,9^ Histopathological examinations reveal, in the early stages, massive periadnexal and perivascular neutrophilic, lymphocytic, and histiocytic infiltrates; older lesions feature the formation of epithelioid-cell granulomas. The main differential diagnosis is fulminant acne, which presents some striking differences: rosacea fulminans typically occurs in older patients; the lesions remain confined to the face and neck; comedones are absent; and less systemic symptoms are observed. Other differential diagnoses include gram-negative folliculitis, gram-negative acne, and fungal and mycobacterial infections.^9^ Recommended treatment consists of high-potency topical or systemic steroids associated with isotretinoin. Nevertheless, exacerbation of the disease during treatment with oral prednisolone has been reported. Dapsone can be used in cases of therapeutic failure or treatment-resistant cases.^10^ Other treatment options include the same drugs used in the treatment of classical rosacea, such as tetracycline, doxycycline, and minocycline.^8^ In the absence of suitable treatment, localized forms can spread. Once lesions are controlled, no recurrence is observed.^2,6^ Pyoderma faciale is a disfiguring disease with striking psychological, emotional, and social reflexes. We report this rare form of the disease and highlight the importance of early treatment to prevent unsightly scars.^1,8^
